# Structural Basis of Protein Oxidation Resistance: A Lysozyme Study

**DOI:** 10.1371/journal.pone.0101642

**Published:** 2014-07-07

**Authors:** Marion Girod, Quentin Enjalbert, Claire Brunet, Rodolphe Antoine, Jérôme Lemoine, Iva Lukac, Miroslav Radman, Anita Krisko, Philippe Dugourd

**Affiliations:** 1 Université de Lyon, 69622, Lyon, France; 2 Institut des Sciences Analytiques, UMR 5280, CNRS, Université Claude Bernard Lyon 1, Lyon, France; 3 Institut Lumière Matière, UMR 5306, CNRS, Université Claude Bernard Lyon 1, Lyon, France; 4 Mediterranean Institute for Life Sciences, Split, Croatia; 5 Liverpool John Moores University, School of Pharmacy and Biomolecular Sciences, Liverpool, Merseyside, England; 6 INSERM U1001, Faculte de Medecine, Universite R. Descartes Paris-5, Paris, France; Medical University of Vienna, Austria

## Abstract

Accumulation of oxidative damage in proteins correlates with aging since it can cause irreversible and progressive degeneration of almost all cellular functions. Apparently, native protein structures have evolved intrinsic resistance to oxidation since perfectly folded proteins are, by large most robust. Here we explore the structural basis of protein resistance to radiation-induced oxidation using chicken egg white lysozyme in the native and misfolded form. We study the differential resistance to oxidative damage of six different parts of native and misfolded lysozyme by a targeted tandem/mass spectrometry approach of its tryptic fragments. The decay of the amount of each lysozyme fragment with increasing radiation dose is found to be a two steps process, characterized by a double exponential evolution of their amounts: the first one can be largely attributed to oxidation of specific amino acids, while the second one corresponds to further degradation of the protein. By correlating these results to the structural parameters computed from molecular dynamics (MD) simulations, we find the protein parts with increased root-mean-square deviation (RMSD) to be more susceptible to modifications. In addition, involvement of amino acid side-chains in hydrogen bonds has a protective effect against oxidation Increased exposure to solvent of individual amino acid side chains correlates with high susceptibility to oxidative and other modifications like side chain fragmentation. Generally, while none of the structural parameters alone can account for the fate of peptides during radiation, together they provide an insight into the relationship between protein structure and susceptibility to oxidation.

## Introduction

Accumulation of oxidative damage in proteins correlates with aging of animals and man and with cell death [1] since it can cause irreversible and progressive degeneration of almost all cellular functions [1], [2]. Proteins can undergo oxidative modifications in a number of ways: mainly oxidation of sulfur-containing and aromatic amino acid side chains, but also the irreversible protein carbonylation on Arg, Lys, Pro and Thr residues, protein backbone oxidation and, finally, protein fragmentation. However, the total amount of oxidative protein modification is a result of the rate of production of corrosive free radicals and the intrinsic resistance of each individual protein species to oxidative damage. Individual proteins show extreme inequalities in their susceptibility to oxidative damage. Even though variety of folds may differ in their resistance to oxidative stress, perfectly folded proteins are, by large most robust [3]. But, their resistance is readily lost by introducing errors in amino acid sequence that lead to folding errors [3], [4]. Clearly, past research leads to an anticipation of a link between protein resistance to misfolding and resistance to oxidation. The sequence or structure determinants underlying the susceptibility of proteins to oxidative damage are not clear, but the strong tendency of carbonylated sites to cluster together [5] hints to localized structural determinants of oxidability.

Recently, advances in mass spectrometry-based proteomics have allowed large-scale studies to identify oxidized proteins and correlate their influence on protein functional activities for a wide range of proteins [6]. Protein oxidation has been characterized by mass spectrometry and serves as a useful marker for assessing oxidative stress in vivo [7]. More especially, carbonylation research is characterized by the application of numerous protocols and proteomics workflows, which rely mostly on various partially investigated carbonyl-labeling techniques [8]–[15]. Protein oxidation is induced either directly by reactive oxygen species or indirectly by reaction with secondary by-products of oxidative stress. Oxidative changes to proteins can lead to diverse functional consequences, such as inhibition of enzymatic and binding activities, increased susceptibility to aggregation and proteolysis or altered immunogenicity. The two amino acids that are the most prone to oxidative attack are Cysteine (Cys) and Methionine (Met), both of which contain susceptible sulfur atoms. Different targeted mass spectrometry approaches have been described for the quantification of protein oxidation, which in case of Cys, leads to the formation of disulfide bonds, mixed disulfides (e.g., with glutathione), and thiyl radicals [16], [17]. For Met residues, the major product of oxidation under biological conditions, Met sulfoxide, has also been quantified by mass spectrometry [18]–[21].

In the present study we explore the structural basis of protein resistance to oxidation during gamma radiation. As a model protein, we have selected lysozyme, an antimicrobial enzyme that is found in a wide variety of organisms including birds, mammals and insects. The lysozyme of chicken egg white has been most extensively studied. Its structure is characterized by high stability, maintained by three disulfide bonds. Herein, we employed the mass spectrometry approach to describe the differential resistance to gamma radiation-induced modifications with native lysozyme and lysozyme with reduced-alkylated 3 disulfide bridges, as a model misfolded protein. The results obtained on the differential resistance of individual regions of native and misfolded lysozyme were complemented with molecular dynamics (MD) simulations performed on both protein forms. Herein, we employed the mass spectrometry approach to describe the differential resistance to gamma radiation-induced modifications with native lysozyme and lysozyme with reduced-alkylated 3 disulfide bridges, as a model misfolded protein. The results obtained on the differential resistance of individual regions of native and misfolded lysozyme were complemented with molecular dynamics (MD) simulations performed on both protein forms. Amino acid side-chain mobility, their involvement in hydrogen bonding and their solvent accessibility is presented as a combination of structural features that best describes the differential susceptibility of protein parts to gamma radiation-induced modifications. While mass spectrometry has been used by several authors to study *in vivo* and *in vitro* protein oxidation, this is to our knowledge the first study that addresses protein radiation resistance by by associating mass spectrometry and molecular dynamics simulations, an approach that could be applied to other systems.

## Materials and Methods

### Chemicals and reagents

Acetonitrile (ACN), methanol (MeOH) and water (LC–MS grade) were obtained from Fisher Scientific (Strasbourg, France). Dithiothreitol (DTT), iodoacetamide (IAM), formic acid (FA) (LC–MS grade), trypsin (type IX-S from Porcine Pancreas), ammonium bicarbonate (AMBIC), ammonium acetate (AA) were purchased from Sigma–Aldrich (St Quentin-Fallavier, France). Chicken lysozyme was purchased from Sigma–Aldrich (St Quentin-Fallavier, France).

### Instrumentation

Discovery experiments were performed on an hybrid quadrupole-orbitrap Q-Exactive mass spectrometer (Thermo Fisher Scientific, San Jose, CA, USA) equipped with a ESI ion source coupled to a Surveyor HPLC-MS pump (Thermo Fisher Scientific, San Jose, CA, USA) and a PAL Auto-sampler (CTC Analytics, Switzerland).

Quantitative analyses were performed on an 4000 QTRAP mass spectrometer (AB Sciex, Foster City, CA, USA) equipped with a Turbo V ion source coupled to an Agilent 1290 series high pressure liquid chromatography (Agilent technologies, Waldbronn, Germany).

### Sample Preparation

Lyophilized lysozyme was dissolved in 10 mM PBS pH 7.4 to a final concentration of 1 mg/mL and irradiated as described below. A separate lysozyme solution was prepared at a final concentration of 3 mg/mL and reduced in 6 M urea, 15 mM dithiothreitol at 60°C for 40 min and then alkylated with 35 mM iodoacetamide at room temperature in dark for 40 min. Final concentration of the reduced-alkylated lysozyme was also 1 mg/mL.

**Figure 1:**
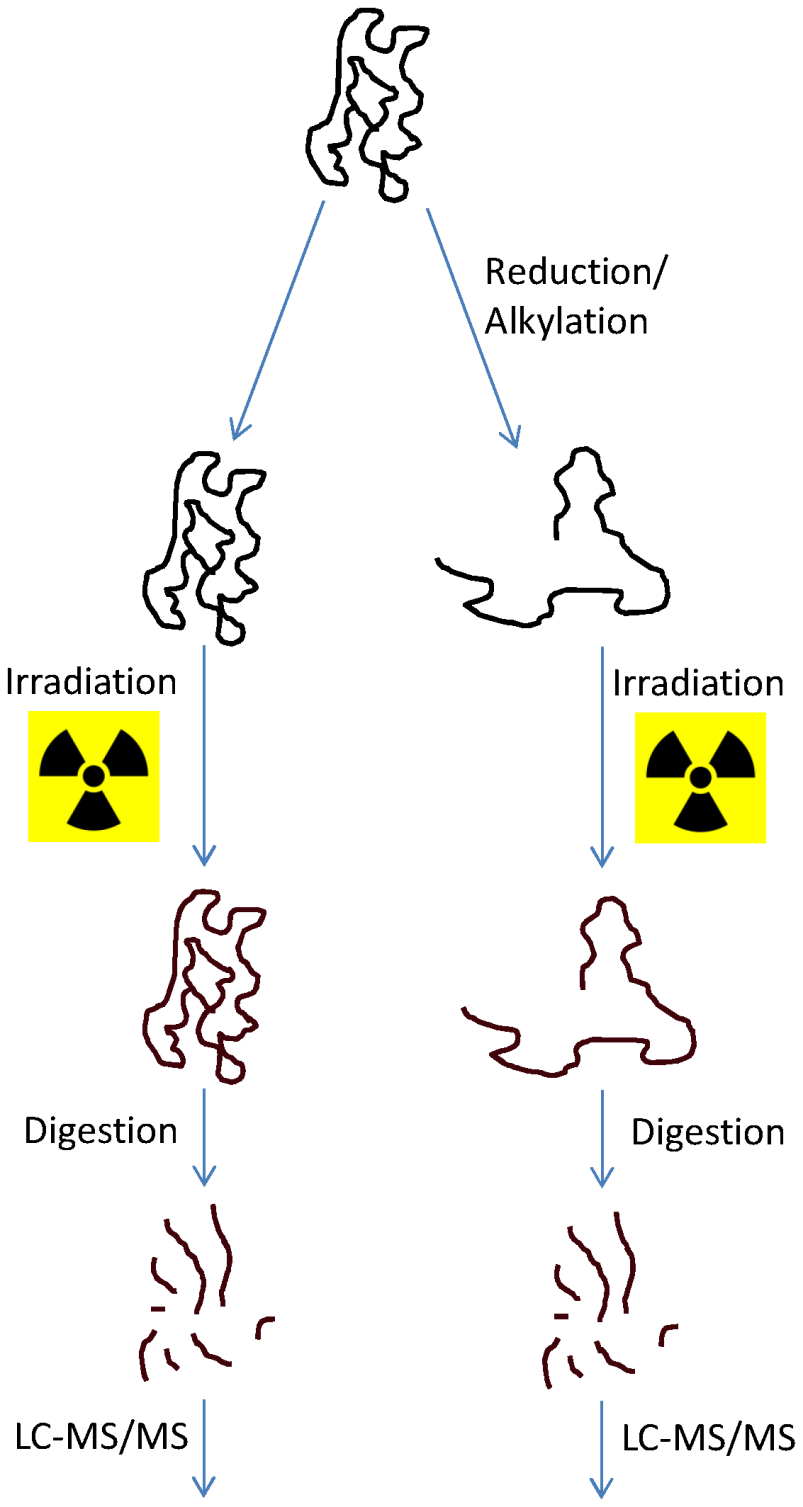
Workflow of the analytical method.

1 mL of native and reduced samples were then irradiated in Eppendorf tubes on ice by ^137^Cs source of gamma radiation to final doses of 10, 20, 50, 100, 200, 500, 1000, 2000 and 5000 Gy (see workflow in Figure 1). In order to reduce the urea concentration, the samples were diluted 5-fold with ammonium bicarbonate prior to overnight digestion at 37°C with trypsin using a 1∶30 (w/w) enzyme to substrate ratio. Digestion was stopped by addition of formic acid to a final concentration of 0.5%.

All samples were desalted and concentrated using Oasis HLB 3 cc (60 mg) reversed phase cartridges (Waters, Milford, MA, USA). Before loading the tryptic digest onto the Oasis cartridges, all cartridges were conditioned with 1 mL of MeOH and then 1 mL of water containing 0.5% FA. After the loading, all cartridges were washed with 1 mL of MeOH/water (5/95, v/v) containing 0.5% FA and eluted with 2 mL of ACN containing 0.5% FA. All samples were evaporated to dryness and resuspended in 100 µL of water/MeOH (2∶1, v/v) containing 0.5% FA. All solutions were stored at −18°C prior to use.

### HPLC separation

For discovery and quantitative analysis, the HPLC separation was carried out on a XBridge C_8_ column (100×2.1 mm, 3.5 µm) from Waters. The HPLC mobile phase consisted of water containing formic acid 0.1% (v/v) as eluent A and ACN containing formic acid 0.1% (v/v) as eluent B. Elution was performed at a flow rate of 300 µL/min with an analysis time of 26 min. The elution sequence included a plateau with 95% of eluent A for 5 min followed by a linear gradient from 98% to 70% over 18 min, then a plateau at 0% of eluent A for 4 min. The gradient was returned to the initial conditions and held there for 3 min. The injection volume was 20 µL.

### Mass Spectrometry Operating Conditions

For discovery analysis, ionization was achieved using electrospray in the positive ionization mode with an ion spray voltage of 4 000 V. The sheath gas and the auxiliary gas (nitrogen) flow rates were respectively set at 35 and 10 (arbitrary unit) with a HESI vaporizer temperature of 410°C. The ion transfer capillary temperature was 300°C with a sweep gas (nitrogen) flow rate at 5 (arbitrary unit). The S-lens RF was set at 90 (arbitrary unit). The orbitrap resolution was 65 000. The Automatic Gain Control (AGC) target was 3e6 and the maximum injection time was set at 250 ms. Experiments were done in data-dependent top 10 mode. The full MS scans were done over a *m/z* 250–1200 range with a resolution of 35 000. For the data-dependent MS/MS scans, the resolution was set at 17 500 with a normalized collision energy of 28 (arbitrary unit).

For quantitative analysis, ionization was achieved using electrospray in the positive ionization mode with an ion spray voltage of 5 500 V. The curtain gas flow (nitrogen), the ion source gas 1 and 2 (nitrogen) were respectively set at 50, 40 and 40 units. The Turbo ion spray source was operating at 500°C. The declustering potential and collision cell exit potential were set at 80 V and 15 V. Q1 and Q3 quadrupole resolutions were adjusted at ∼1.1±0.1 amu. 24 SRM transitions (couple precursor ion/product ion) were followed in SRM experiments with a dwell time of 50 ms (see Table 1). For CID experiments the collision energy was set to 28 eV for doubly charged ions.

**Table 1:** Summary of the data for observed peptides analyzed by SRM.

Peptide (and position in the protein sequence)	Transitions (Q1/Q3)	Fragment ion
**CELAAAMK** (6–13)	447.21/491.26	(y5)^+^
	447.21/604.35	(y6)^+^
	447.21/290.08	(b2)^+^
**HGLDNYR** (15–21)	437.71/680.34	(y5)^+^
	437.71/737.36	(y6)^+^
	437.71/195.09	(b2)^+^
**GYSLGNWVCAAK** (22–33)	663.32/905.43	(y8)^+^
	663.32/1105.55	(y10)^+^
	663.32/221.09	(b2)^+^
**FESNFNTQATNR** (34–45)	714.83/951.46	(y8)^+^
	714.83/1152.54	(y10)^+^
	714.83/277.12	(b2)^+^
**WWCNDGR** (62–68)	497.20/232.14	(y2)^+^
	497.20/621.24	(y5)^+^
	497.20/807.32	(y6)^+^
**GTDVQAWIR** (117–125)	523.27/673.38	(y5)^+^
	523.27/887.47	(y7)^+^
	523.27/159.08	(b2)^+^
**CELAAA** ***M*** **K** (6–13)	455.21/507.26	(y5)^+^
	455.21/620.34	(y6)^+^
	455.21/290.08	(b2)^+^
**GTDVQA** ***W*** **IR** (117–125)	531.27/689.37	(y5)^+^
	531.27/788.44	(y6)^+^
	531.27/159.08	(b2)^+^
**NTDGSTDYGILQINSR** (46–61)	Not followed in SRM	-
**IVSDGNGMNAWVAWR** (98–112)	Not followed in SRM	-

### Molecular dynamics simulations

Molecular dynamics (MD) simulations of the native and reduced lyzozyme were performed with Gromacs 4.5.5 [22] package using Amber99sb force field. Protein (PDB: 1AKI) was placed into cubic boxes filled with water molecules. Solvent molecules were modelled using TIP3P water model. To ensure that the system has no steric clashes or inappropriate geometry, it was minimized by 50 000 steps of the steepest descent minimization. Counterions were added to ensure a net neutral charge and preequilibration step was run.

Simulation snapshots were collected after every 2 ps of 30 ns long simulation, resulting in 15000 snapshots for every run. Temperature and pressure were kept constant at standard conditions (10^5^ Pa and 300 K), using temperature coupling with modified Berendsen thermostat with coupling constant of 0.1 ps, and Parinello-Rahman barostat with 2 ps coupling constant. All bonds were constrained using LINear Constraint Solver (LINCS) procedure [23]. Leap-frog integrator with 2 fs timestep was used and maximum number of steps was 5000000. Long range dispersion corrections for energy and pressure were used. Snapshots were superimposed based on root-mean square deviation (RMSD) of C_á_ atoms using VMD [24]. Solvent accessibility surface area (SASA) was calculated using Gromacs g_sas function. Hydrogen bonds were found using Gromacs g_hbond function and the percentage of time that a given hydrogen bond existed during the trajectory was calculated. Next, a Perl script was utilized that summed these percentages per lysozyme fragment where the input is the existence matrix for all hydrogen bonds over all frames. A sum of such percentages of time of each hydrogen bond existence per lysozyme fragment is called occupancy and it is computed for each lysozyme fragment monitored herein. Sidechain-sidechain and sidechain-backbone hydrogen bonds were further analyzed. In order to compute dG_solv_ Gromcs g_sas function was used based on Eisenhaber *et al*. work [25]. Structures with reduced disulfide bonds were prepared using MAESTRO 9.3 [26], by mutating Cys94, Cys76, Cys80, Cys64, Cys115, Cys30, Cys6 and Cys127 to Gly, a replacement standardly used in MD simulations [27], [28].

## Results and Discussion

### Mass spectrometry analysis of lysozyme radiation resistance

**Figure 2:**
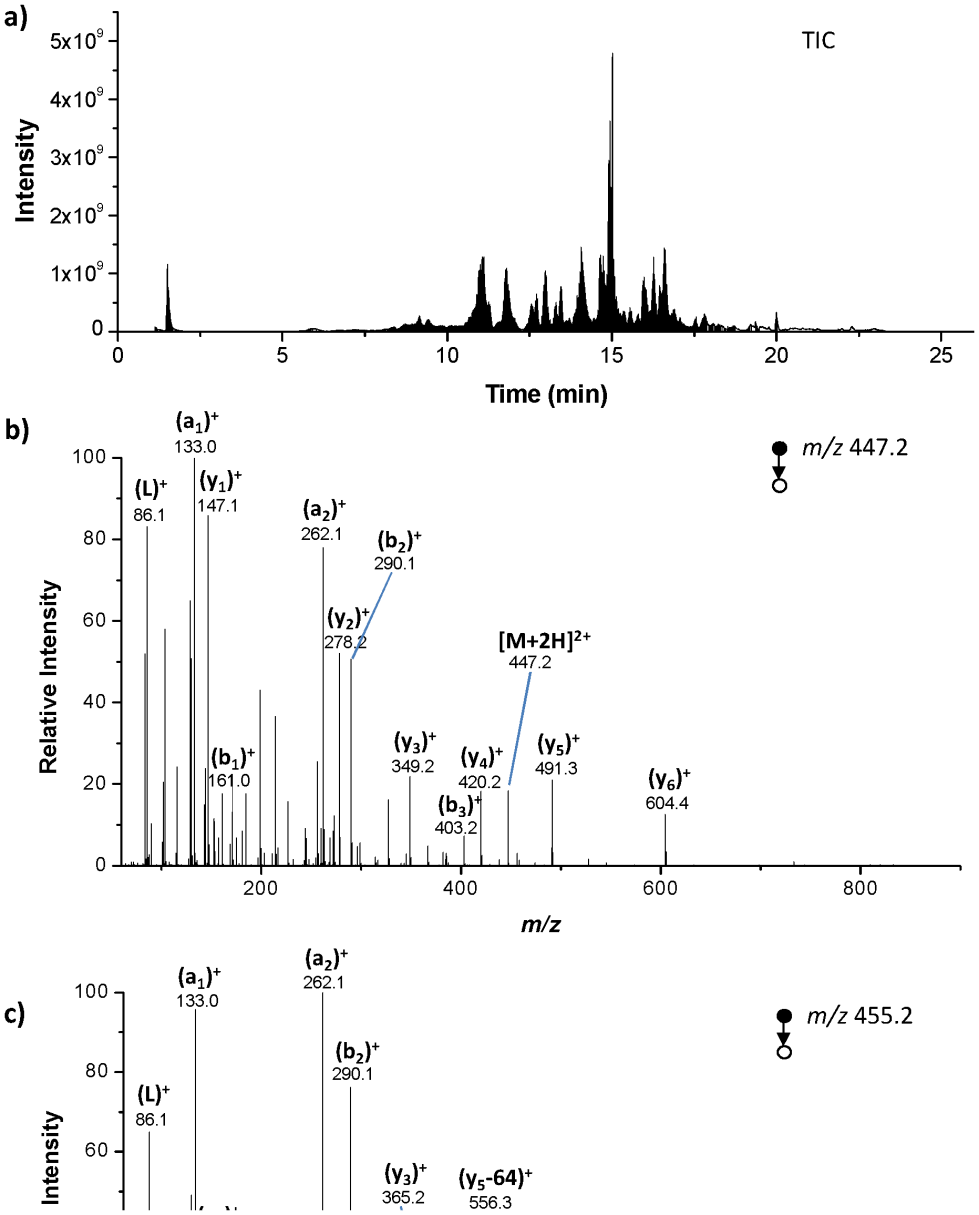
Analysis of the reduced-alkylated lysozyme irradiated with a dose at 1000 Gy. (a) TIC chromatogram, (b) CID spectrum of the CELAAAMK ion *m/z* 447.2 detected between 8.8 and 9.9 min and (c) CID spectrum of the CELAAA*M*K ion *m/z* 455.2 detected between 6.8 and 7.2 min.

In order to facilitate the location of oxidative modifications of native and reduced-alkylated lysozyme after gamma irradiation, a bottom-up proteomic strategy was applied. Trypsin hydrolysate of native and reduced irradiated forms were analyzed by HPLC coupled to high-resolution mass spectrometer in a data dependant acquisition mode (top 10 experiment) in order to obtain for each peptide both the accurate mass and sequence information deduced from their MS/MS spectra. As an example, Figure 2a shows the TIC chromatogram for the analysis of the reduced-alkylated lysozyme irradiated with a dose of 1000 Gy. Raw data were analyzed by Protein Prospector database searching tool to assign peptide sequences according predicted *b* and *y* fragment ions. Figure 2b displays the CID spectrum of the ion at *m/z* 447.2 detected between 8.8 and 9.9 min. The *b* and *y* fragment ion pattern has been attributed to doubly charged non-oxidized CELAAAMK lysozyme tryptic fragment. The CID spectrum of the ion at *m/z* 455.2, detected between 6.8 and 7.2 min, (Figure 2c) shows a shift of +16 mass units from y_2_ to y_6_ fragment ions, which is characteristic of the presence of a mono oxidized methionine form in CELAAAMK peptide. This hypothesis is strengthened by satellite peaks corresponding to neutral loss of -64 from y_2_ to y_6_ (noted y-64 in Figure 2c). They result from the elimination of a HSO-CH_3_ group from the oxidized side chain of methionine residue (Met12).

Following Protein Prospector analysis, 8 normal peptides and 2 oxidized peptides were detected and validated (Table 1), which corresponds to a sequence coverage of 66.6%. Oxidation events were observed on Met12 residue for the CELAAA*M*K peptide as well as on Trp123 residue for the GTDVQA*W*IR peptide (+16 mass units). The same 10 peptides were identified for both the native and reduced forms of the protein.

**Figure 3:**
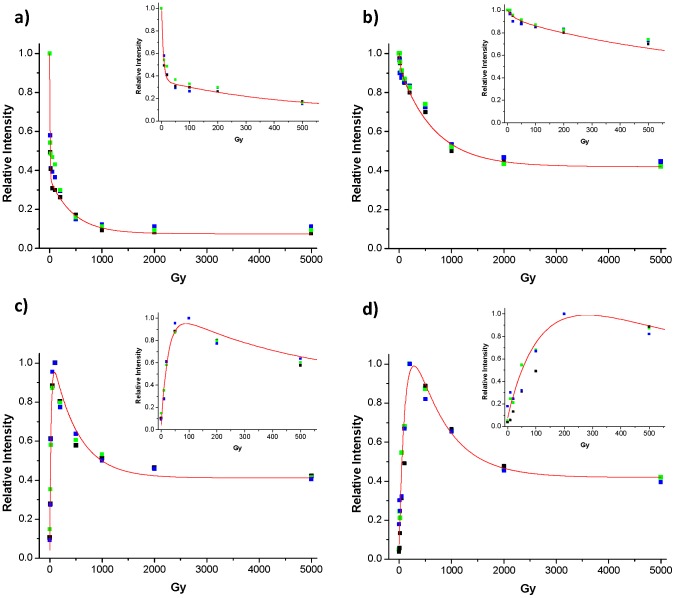
Evolution of the signal of the CELAAAMK peptide (a, b) and oxidized CELAAA*M*K peptide (c, d). CELAAAMK peptide (3 transitions) irradiated in the native (a) and reduced-alkylated form (b) Oxidized CELAAA*M*K peptide (3 transitions) irradiated in the native (c) and reduced-alkylated form (d).

Next, the relative quantifications of non-oxidized and oxidized lysozyme peptides were performed as a function of irradiation dose for both the native and reduced-alkylated irradiated lysozyme in Selected Reaction Monitoring mode (SRM). The peptides NTDGSTDYYGILQINSR and IVSDGNGMNAWVAWR were not followed because of possible errors in the transitions specificity due to the long sequences (>12 amino acids). Several transitions were monitored for each tryptic peptide target and are presented in Table 1. The evolution of each peptide population, measured as the area under the chromatogram peak for each transition, is plotted as a function of the irradiation dose. The amount of non-oxidized peptides (expressed as the chromatogram intensity) decreases as the gamma irradiation dose increases. Figure 3a and 3b show the evolution of the signal of the irradiated CELAAAMK peptide (3 transitions) from the native and reduced-alkylated lysozyme, respectively. The curves display the kinetics of decrease in the amount of peptide with two characteristic times. The use of a double exponential decay equation (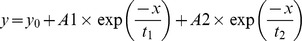) to fit the experimental data leads to two doses for the peptide half-life within the native protein: t_1_ = 7.97 Gy and a higher one t_2_ = 445.7 Gy. The first value is partially attributed to the oxidation of the protein (vida infra), while the second one corresponds to further degradation of the protein. This peptide is more resistant to radiation in the reduced-alkylated form of lysozyme relative to the native one if we compare the results for the native form (a) and the reduced form (b). Indeed, the first half-life dose of oxidation kinetics is higher for the CELAAAMK peptide coming from the reduced irradiated protein (t_1_ = 44.5 Gy).

The concentration of the peptide CELAAA*M*K with the oxidized Met12 residue follows a different behavior. Figure 3c and 3d show the signal evolution of the oxidized CELAAA*M*K peptide (3 transitions), irradiated in the native and reduced-alkylated lysozyme, respectively. The signal representative of the oxidized peptide concentration increases from 0 to 100 Gy followed by a decrease in concentration. Using a double exponential growth and decay equation (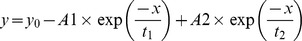) to fit the data, we can observe two phenomena: (I) the kinetics of formation of the oxidized methionine species (t_1_ = 25.1 Gy for the native irradiated form) which corresponds to the oxidation of the native protein and, (II) the degradation of the oxidized species (t_2_ = 485.2 Gy for the native irradiated form). A difference in the kinetics of oxidized peptide formation in the native and the reduced-alkylated protein is observed upon irradiation. The formation of the oxidized peptide CELAAA*M*K is slower in the case of the reduced-alkylated irradiated lysozyme (t_1_ = 44.5 Gy), than for the native lysozyme (t_1_ = 25.1 Gy). This is consistent with the t_1_ measured for CELAAAMK, which is lower for the reduced form than the native one. The difference in the half-life doses observed for the degradation (fast decay value) of the normal peptide and formation of the oxidized peptide suggests that the oxidation of the methionine is not the only process occurring at low doses.

Table 2 summarizes characteristic t_1_ and t_2_ doses determined for all analyzed peptides. From triplicate analysis, we were able to calculate imprecision values expressed by the coefficient of variation (CV), which were all better than 20%. This validates that the robustness and reproducibility of the analytical procedure is validated. While the t_1_ value seems to strongly depend on the nature of tryptic peptide (i.e. sequence and structure), t_2_ values are all in the same order of magnitude. This indicates that the fastest of the overlapping processes, which is at least in part attributed to oxidation, depends on the sequence and structure of the protein, while further degradation is a generalized process that is less sensitive to the sequence and structure of the protein.

**Table 2:** Characteristic doses t_1_ and t_2_ for observed peptides from native and reduced-alkylated form of lysozyme.

Peptide	From Native irradiated Lysozyme				From Reduced-alkylated irradiated Lysozyme			
	t_1_ (Gy)	CV (%, *n* = 3)	t_2_ (Gy)	CV (%, *n* = 3)	t_1_ (Gy)	CV (%, *n* = 3)	t_2_ (Gy)	CV (%, *n* = 3)
CELAAAMK	7.9	9	445.7	10	44.5	19	670.2	12
CELAAA*M*K	25.1	18	485.2	17	75.8	9	641.1	6
GTDVQAWIR	58.9	14	338.9	15	21.1	14	570.3	10
GTDVQA*W*IR	82.4	10	675.2	8	38.9	16	651.6	15
HGLDNYR	50.2	4	375.2	6	18.5	17	386.7	19
GYSLGNWVCAAK	88.8	13	279.2	14	17.3	6	346.3	4
FESNFNTQATNR	19.4	7	549.1	9	11.5	6	502.6	9
WWCNDGR	75.4	15	654.2	12	33.8	12	678.6	8

CV stands for coefficient of variation.

Furthermore, Table 3 presents the doses for which the peptide amount (intensity) is half of the initial one at dose 0 (D_50_) for all analyzed peptides. According to this parameter, peptides WWCNDGR and GTDVQAWIR are the most resistant peptides in the native lysozyme with D_50_ of 510 Gy and 385 Gy, respectively. An intermediate resistance is observed for peptide GYSLGNWVCAAK with D_50_ of 150 Gy. Finally, the most sensitive peptides are HGLDNYR, FESNFNTQATNR and CELAAAMK with D_50_ of 50 Gy, 30 Gy and 10 Gy, respectively.

**Table 3:** Half-life irradiation doses (D_50_) and saturating intensity (I_sat_) measured for each observed peptide from native and reduced-alkylated form of lysozyme.

Peptide	From Native irradiated Lysozyme				From Reduced-alkylated irradiated Lysozyme			
	D_50_ (Gy)	CV (%, *n* = 3)	I_sat_ (%)	CV (%, *n* = 3)	D_50_ (Gy)	CV (%, *n* = 3)	I_sat_ (%)	CV (%, *n* = 3)
CELAAAMK	10	12	7.5	7	1241	18	42	12
GTDVQAWIR	385	11	28.6	12	26	9	11	11
HGLDNYR	50	7	9.1	8	100	8	12.5	8
GYSLGNWVCAAK	150	8	13.2	7	298	16	32.1	10
FESNFNTQATNR	30	12	8.4	10	10	10	7.1	11
WWCNDGR	510	18	32.6	9	130	8	27.9	12

CV stands for coefficient of variation.

In the reduced-alkylated lysozyme, the resistance pattern of the peptides is rather different: CELAAAMK peptide appears as the most resistant with a D_50_ of 1241 Gy, followed by GYSLGNWVCAAK, WWCNDGR and HGLDNYR peptides with D_50_ of 298 Gy, 130 Gy and 100 Gy, respectively. The most sensitive peptides are GTDVQAWIR and FESNFNTQATN with D_50_ of 26 Gy and 10 Gy, respectively.

Clearly, the conformational change of lysozyme induced by the reduction and alkylation of the three disulfide bridges did not yield the same effect on all parts of the protein. Peptides CELAAAMK, HGLDNYR and GYSLGNWVCAAK showed an increase in the radiation resistance upon the conformational change triggered by the disulfide reduction. Moreover, the most sensitive CELAAAMK peptide in the native form of lysozyme became the most resistant protein fragment in the reduced-alkylated form. On the other hand, the same conformational change rendered GTDVQAWIR, FESNFNTQATN and WWCNDGR peptides more sensitive with respect to the native form. Note that the CVs of the half-life values are also better than 20% reflecting reproducible measurements (Table 3). It should be pointed out that D_50_ correlates with the t_1_ values reported in Table 2: both parameters, therefore, report on the radiation resistance of the peptides.

In this context, we also measured the value of intensity at which the amount of the non-oxidized peptide saturates at its minimum, i.e. does not decrease further (saturating intensity, I_sat_, Table 3). This value reports on the remaining amount of each peptide that has not experienced any modifications, even at highest applied doses of gamma radiation. Namely, the peptides that displayed the highest resistance according to D_50_ are also characterized by the largest proportion (24.8%, in average) of the remaining non-modified peptide (not affected by radiation) at 5000 Gy (GTDVQAWIR, WWCNDGR and GYSLGNWVCAAK). On the other hand, CELAAAMK, HGLDNYR and FESNFNTQATN peptides displayed the lowest remaining amount of the non-modified peptide with the average of 8.3%. In the reduced-alkylated lysozyme, CELAAAMK, HGLDNYR, GYSLGNWVCAAK and WWCNDGR peptides display an increase in the I_sat_ relative to the native protein, indicating their increased resistance due to the conformational change. GTDVQAWIR and FESNFNTQATN peptides show a decreased I_sat_ in the reduced-alkylated form with respect to the native one. To summarize, I_sat_ is another measure of peptide radiation resistance that correlates well with D_50_ (Table 3).

### Structural basis of differential radiation resistance within lysozyme

Why do different parts of lysozyme display different resistance to detrimental effects of gamma radiation?

**Figure 4:**
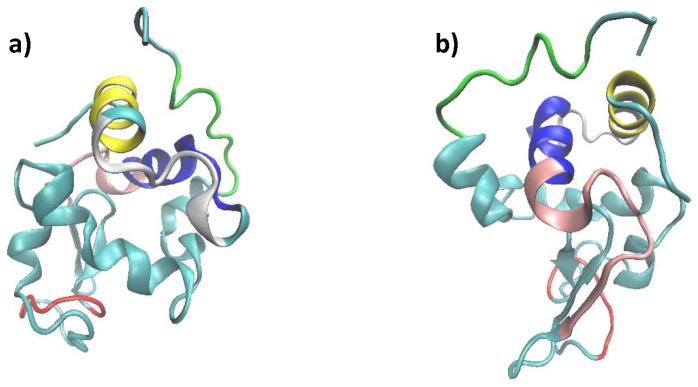
Lysozyme tertiary structure (PDB:1AKI). (a) CELAAAMK is shown in yellow, GTDVQAWIR in green, HGLDNYR in white, GYSLGNWVCAAK in blue, FESNFNTQATNR in pink and WWCNDGR in red. (b) 180° degree rotation around y-axis.

**Figure 5:**
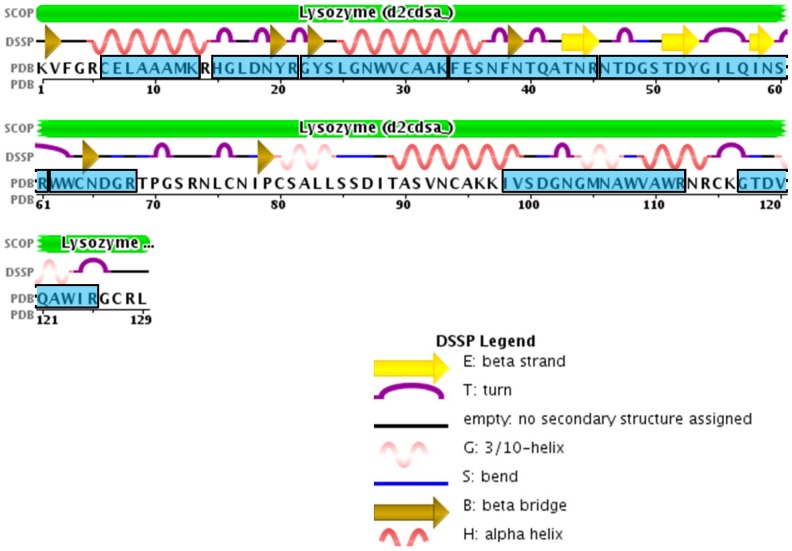
Sequence of the chicken lysozyme with, highlighted in blue, the detected peptides.

Based on the tertiary structure of lysozyme (Figure 4), the peptides with highest sensitivity in the native protein (CELAAAMK, HGLDNYR and FESNFNTQATN) are organized in very different secondary structures. While CELAAAMK is folded into an alpha-helix, the other two peptides seem to be folded into a combination of short beta-sheets and beta-turns (Figure 5). On the other hand, the most resistant peptides are folded into 3/10 helix (GTDVQAWIR), alpha helix (GYSLGNWVCAAK) and a combination of beta strand and loop structure (WWCNDGR). Since no particular trend can be observed in the context of peptides' secondary structures, such analysis prevents us from drawing any definitive conclusion about the influence of secondary structure on protein radiation resistance.

Therefore, we performed a series of MD simulations of both native and reduced-alkylated forms of lysozyme. Several structural parameters have been computed during the simulations and compared with the peptide radiation resistance.

The root-mean-square deviation (RMSD) of atomic positions reflects the flexibility and dynamics of the peptide fragments inside the protein. We hypothesized that a part of the protein that is characterized by high flexibility would also be more available for modifications by toxic products of gamma radiation. Herein, we have computed average RMSD for backbone and side-chains of each peptide observed in the mass spectrometry analysis. The results are summarized in Table 4.

**Table 4:** Summary of backbone and side-chain root mean square deviation (RMSD) data for each observed peptide from the native and reduced-alkylated lysozyme.

	Average RMSD Å							
	Native, run 1		Native, run 2		Reduced-alkylated, run 1		Reduced-alkylated, run 2	
	Backbone	Side-chain	Backbone	Side-chain	Backbone	Side-chain	Backbone	Side-chain
CELAAAMK	0.79	1.25	0.83	1.22	0.78	1.37	1.06	1.47
GTDVQAWIR	1.03	1.65	1.21	1.56	1.42	1.94	1.44	1.9
HGLDNYR	1.03	1.94	1.02	2.01	1.14	1.98	1.2	2.02
GYSLGNWVCAAK	0.66	0.81	0.83	1.11	0.94	1.21	0.87	1.15
FESNFNTQATNR	0.77	1.11	1.22	2.64	0.93	1.6	0.87	1.73
WWCNDGR	0.74	1.3	0.92	1.53	1.34	2.63	1.31	2.24

The results of the two molecular simulation runs are presented separately.

The side-chain average RMSD of the three most sensitive peptides (CELAAAMK, HGLDNYR and FESNFNTQATNR) is 1.69 Å, while the three most resistant ones (GTDVQAWIR, WWCNDGR and GYSLGNWVCAAK) display an average side-chain RMSD of 1.32 Å. Although inter-peptide differences exist also in backbone RMSD, they could not be related to the differences in their D_50_ (Table 3). On the other hand, in the reduced-alkylated form of lysozyme, such correlation could not be observed among the fragments: while the most resistant peptides display backbone and side-chain RMSD of 1.05 and 1.75, respectively, in case of the sensitive peptides the values are 1.17 and 1.79 (Table 4).

The comparison of fragments between native and reduced-alkylated forms of lysozyme resulted in the following observations: the peptides that display a decrease in D_50_ upon the reduction of disulfide bridges (GTDVQAWIR and WWCNDGR), also show an increase in RMSD of both backbone and side-chain. In the case of GTDVQAWIR peptide, average backbone RMSD increased from 1.12 Å to 1.43 Å; side-chain RMSD increased from 1.6 Å to 1.92 Å. Average backbone RMSD of WWCNDGR peptide increased from 1.41 Å in the native protein to 2.43 Å in the reduced-alkylated form of lysozyme. Average side-chain RMSD of the same peptide increased from 0.83 Å to 1.32 Å upon disulfide reduction. The three most resistant peptides, whose D_50_ increases upon disulfide reduction do not display significant changes in their RMSD. Based on these results, it can be proposed that a higher side-chain mobility could contributes to, but is not sufficient to account for the increased susceptibility to modifications during gamma radiation.

Furthermore, we computed the occupancy of side-chain—backbone and side-chains—side-chain hydrogen bonds of each fragment (description in the Methods section). While backbone hydrogen bonds involvement does not show any trend between fragments, the situation is different for the side-chain hydrogen bonds (Table 5). Namely, the three most resistant peptides from native lysozyme have in average 132% occupancy of H-bonds while the sensitive ones have 85%. In the reduced-alkylated form of lysozyme, the most resistant peptides have an average of 118% occupancy of H-bonds, while the sensitive ones have 53%. In comparison between the native and the reduced-alkylated lysozyme, two peptides display large changes in H-bond occupancy. Namely, in FESNFNTQATNR peptide the occupancy of H-bonds decreases from 643% to 560%, and the side-chain ones from 134% to 33%. However, WWCNDGR peptide experiences an increase of side-chain H-bond occupancy from 59% to 102%. Interestingly, the resistance of both of these peptides drops upon reduction of disulfide bonds. In spite of the special case of the WWCNGDR peptide (discussed further below), these results lead to the conclusion that the involvement of side-chains in H-bonds may be protective against their modification during gamma radiation.

**Table 5:** Average number of occupied hydrogen bonds in % (total and side-chain—side-chain) for each observed peptide from the native and reduced-alkylated lysozyme.

	Hydrogen bonds							
	Native, run 1		Native, run 2		Reduced-alkylated, run 1		Reduced-alkylated, run 2	
	Total	Side-chain	Total	Side-chain	Total	Side-chain	Total	Side-chain
CELAAAMK	588	120	614	114	530	98	546	106
GTDVQAWIR	202	14	193	7	165	3	212	1.6
HGLDNYR	562	88	563	74	573	93	544	55
GYSLGNWVCAAK	758	145	529	124	590	33	531	33
FESNFNTQATNR	640	44	860	75	1142	122	923	83
WWCNDGR	833	299	891	205	777	208	847	322

The results of the two molecular simulation runs are presented separately.

In order to find additional parameters that may explain the behavior of peptides during gamma radiation, we sought for solvent-accessible surface area (SASA), representing the surface accessible to the water. This parameter was computed for those amino-acid side-chains that are sensitive to oxidative modifications, i.e. methionine and tryptophan. The results of their RMSD and SASA are summarized in Table 6. The Trp62 and Trp63 residues (from the WWCNDGR peptide) display interesting behavior: in the native lysozyme Trp62 displays a higher RMSD than Trp63. Furthermore, RMSD of Trp63 also increases in the reduced-alkylated protein form relative to the native one. Such behavior is paralleled with the changes in SASA of the hydrophobic parts of the Trp62 and Trp63 side-chains which increased in the reduced-alkylated lysozyme relative to the native one. These results could explain the deviant behavior of the WWCNDGR peptide. Namely, even though hydrogen bond number increases for this fragment in the reduced-alkylated form relative to the native one, the peptide becomes more sensitive by the conformation change.

**Table 6:** Summary of root mean square deviation (rmsd) and solvent accessible surface area (SASA) for Trp 62, Trp63 and Trp123 residues from the native and reduced-alkylated lysozyme.

	Trp62 (**W**WCNDGR)				
	RMSD	SASA (nm^2^)			
		hydrophobic	hydrophilic	total	ÄG_solv_ (kJ/mol)
Native, run 1	1.26	0.93	0.13	1.05	−2.65
Native, run 2	0.96	0.97	0.13	1.10	−2.77
Reduced-alkylated, run 1	1.07	1.07	0.12	1.20	−3.00
Reduced-alkylated, run 2	1.09	1.09	0.13	1.22	−3.06
	Trp63 (W**W**CNDGR)				
	RMSD	SASA (nm^2^)			
		hydrophobic	hydrophilic	total	ÄG_solv_ (kJ/mol)
Native, run 1	0.64	0.35	0.09	0.44	−1.11
Native, run 2	0.74	0.30	0.10	0.40	−1.01
Reduced-alkylated, run 1	1.41	0.45	0.11	0.57	−1.42
Reduced-alkylated, run 2	1.53	0.38	0.09	0.47	−1.17
	Trp123 (GTDVQA**W**IR)				
	RMSD	SASA (nm^2^)			
		hydrophobic	hydrophilic	total	ÄG_solv_ (kJ/mol)
Native, run 1	1.06	0.52	0.13	0.65	−1.64
Native, run 2	1.21	0.47	0.11	0.58	−1.47
Reduced-alkylated, run 1	1.4	0.47	0.13	0.60	−1.52
Reduced-alkylated, run 2	1.92	0.58	0.10	0.68	−1.72

The results of the two molecular simulation runs are presented separately.

In addition Trp123, which belongs to the GTDVQAWIR peptide, also shows an increase in RMSD in the reduced-alkylated lysozyme relative to the native one (Table 6). This result is consistent with the increased sensitivity of this peptide to gamma radiation, accompanied also by an increase in its side-chain and backbone RMSD.

Previous research has attempted to reveal the relationship between local protein structure and its radiation resistance. Rao and Moller have investigated the appearance of protein carbonylation sites and found a very strong tendency of such sites to cluster together in the protein primary sequence. Furthermore, solvent accessibility has been discussed as a likely determinant of carbonylation-prone sites [29]. Our study presents an original approach that relies on the combination of experimental mass spectrometry based and theoretical molecular dynamics approaches. While none of the analyzed structural parameters alone can explain the differential radiation resistance within different regions of lysozyme, it can be concluded that together they provide an insight into the relationship between protein structure determinants and protein radiation resistance.

## Conclusion

In conclusion, we studied the radiation resistance of six regions of lysozyme in the native and reduced-alkylated form of the protein. We described their differential resistance via two main parameters: the dose that leaves 50% of the native peptide (D_50_) and the amount of the native peptide remaining even at the highest doses of radiation (I_sat_). The decay of the amount of each peptide is a two steps mechanism, characterized by a double exponential evolution of their amounts: the first one is at least partially attributed to oxidation of specific amino acids, while the second one corresponds to further degradation of the protein.

By confronting these results to the structural parameters computed from MD simulations, we were able to conclude that protein parts with increased RMSD are more susceptible to modifications. In addition, involvement of amino acid side-chains in a large number of hydrogen bonds may have a protective effect against modifications. Finally, at the level of individual amino acid side chains we were able to observe that their increased exposure to solvent renders them highly susceptible to oxidative and other modifications like side chain fragmentation. Generally, none of the structural parameters alone is able to account for the behavior of peptides during radiation, however, when combined they provide a valuable insight into the relationship of protein structure and susceptibility to oxidation.
